# Bovine Viral Diarrhea Virus-1 (*Pestivirus bovis*) Associated with Stillborn and Mummified Fetuses in Farmed White-Tailed Deer (*Odocoileus virginianus*) in Florida

**DOI:** 10.3390/v17081104

**Published:** 2025-08-12

**Authors:** An-Chi Cheng, Emily DeRuyter, Pedro H. de Oliveira Viadanna, Zoe S. White, John A. Lednicky, Samantha M. Wisely, Kuttichantran Subramaniam, Juan M. Campos Krauer

**Affiliations:** 1 Department of Large Animal Clinical Sciences, College of Veterinary Medicine, University of Florida, Gainesville, FL 32608, USA; anchicheng@ufl.edu; 2 Department of Environmental and Global Health, College of Public Health and Health Professions, University of Florida, Gainesville, FL 32610, USA; emilyderuyter@ufl.edu (E.D.); jlednicky@phhp.ufl.edu (J.A.L.); 3 Emerging Pathogens Institute, University of Florida, Gainesville, FL 32610, USA; pedro.viadanna@wsu.edu (P.H.d.O.V.); wisely@ufl.edu (S.M.W.); kuttichantran@ufl.edu (K.S.); 4 Department of Infectious Diseases and Immunology, College of Veterinary Medicine, University of Florida, Gainesville, FL 32608, USA; 5 Department of Wildlife Ecology and Conservation, Institute of Food and Agricultural Sciences, University of Florida, Gainesville, FL 32611, USA; zoezy5@gmail.com

**Keywords:** bovine viral diarrhea virus, BVDV, *Pestivirus bovis*, white-tailed deer, deer farming, fetal mummification

## Abstract

Bovine viral diarrhea virus (BVDV) is a globally significant pathogen affecting both domestic livestock and wildlife, including white-tailed deer (WTD; *Odocoileus virginianus*). While experimental infections have demonstrated WTD susceptibility to BVDV, natural infections and associated reproductive outcomes remain scarcely documented. Here, we report the first confirmed case of naturally occurring BVDV-1 infection associated with fetal mummification in farmed WTD in Florida. A two-year-old doe experienced a stillbirth involving two mummified fetuses, which were submitted for necropsy and laboratory diagnostics. Gross findings included diarrhea and underdeveloped eyes in the fetuses, along with small white nodules indicative of amnion nodosum. While not harmful, this condition suggests underlying fetal compromise or intrauterine stress. Virus isolation using Vero E6 and bovine turbinate cell lines, along with a reverse transcription PCR (RT-PCR) assay specifically developed in this study, confirmed the presence of BVDV-1 (*Pestivirus bovis*) RNA in both maternal and fetal samples, suggesting vertical transmission. Sanger sequencing of RT-PCR amplicons further verified the virus species as BVDV-1. Differential diagnostics for other pathogens, including bluetongue virus, epizootic hemorrhagic disease virus, *Mycobacterium* spp., and *Toxoplasma gondii*, were negative. These findings underscore the importance of using biosecurity measures and including BVDV in the differential diagnosis of abortions to reduce the risk of BVDV transmission and potential outbreaks on deer farms, particularly those close to cattle operations. The molecular tools developed in this study provide a robust framework for improved detection and monitoring of BVDV in both wildlife and livestock populations.

## 1. Introduction

Bovine viral diarrhea virus (BVDV) is a single-stranded, positive-sense RNA virus in the genus *Pestivirus* and the family *Flaviviridae*, with a genome length of 12.5 kb. (https://ictv.global/report/chapter/flaviviridaeport/flaviviridaeport/flaviviridae/pestivirus, accessed on 1 June 2025). Bovine viral diarrhea virus-1 (*Pestivirus bovis*, formerly *Pestivirus* A) was first discovered in New York State in the United States of America (USA) at a beef cattle farm in 1946 [1]. In 1994, BVDV-2 (*Pestivirus tauri*, formerly *Pestivirus* B), a new species of BVDV, was discovered [2,3]. Currently, BVDV-1 is prevalent worldwide, whereas BVDV-2 is mainly found in North and South America [4].

The host range for BVDV-1 and -2 includes most animals in the order Artiodactyla, including American bison (*Bison bison*) [5], cattle [6], domestic pigs [7,8], and white-tailed deer (WTD; *Odocoileus virginianus*) [9]. These pathogenic viruses can affect wildlife and present a risk of spillback transmission to livestock. Bovine viral diarrhea viruses-1 and -2 can be transmitted through direct contact, including vertical transmission, and indirect contact through bodily secretions, contaminated fomites, and possibly insect vectors [10,11,12]. Direct and indirect transmission of BVDV between WTD and cattle [13,14], and vertical transmission leading to persistently infected (PI) WTD offspring has been observed in laboratory settings [15].

Previous studies have documented WTD susceptibility to BVDV-1 and -2 infections, resulting in clinical signs such as abortion, bloody vaginal discharge, coughing, depression, lethargy, mummified fetuses, and pyrexia [15,16,17,18,19,20,21]. Experimental transmission of BVDV in WTD during the first or second trimester of gestation resulted in abortion and fetal mummification but not during the third trimester [13,15,17,19,20]. Rather, all does infected during the third trimester gave birth to live, healthy fawns with antibodies against BVDV [20]. The consequences of BVDV infection in WTD during pregnancy are therefore similar to those observed in cattle.

Persistently infected offspring can be produced by cattle and WTD infected with BVDV-1 and -2 during the first trimester, causing the fetuses to develop immune tolerance to the viral proteins [13,15,17,19,22]. Persistently infected animals can spread the virus throughout their lifetime and give birth to PI offspring without presenting any clinical manifestations. Bovine viral diarrhea viruses-1 and -2 can be found in the feces, milk, nasal secretion, saliva, semen, and the urine of PI animals.

BVDV biotypes have been identified based on the ability of the virus to produce cytopathic effects on cultured cells [23,24]. Cytopathic (CP) and non-cytopathic (NCP) are different biotypes found in both BVDV-1 and BVDV-2. Cytopathic biotype causes visible changes in cell culture morphology, such as rounding, detachment, and cell death, while NCP strains do not induce these effects but can still be detected from infected cells using molecular testing or immunological methods [25]. Cytopathic biotypes arise from NCP biotypes because of genetic alterations, while NCP biotypes are more commonly found in nature and can cause persistent BVDV infections in animals [26].

There are limited studies on the prevalence and impact of BVDV-1 and -2 on farmed WTD. Due to the high genetic variability of BVDV and the increasing global transportation of animals, there is a need for comprehensive BVDV surveillance strategies. The clinical manifestations and genomic characteristics of BVDV-1 in farmed WTD in Florida are presented in this case report. The results underscore the critical importance of increasing biosecurity and observational vigilance to mitigate the risk of BVDV transmission and prevent potential BVDV outbreaks at deer farms, especially those that share fences with cattle.

## 2. Materials and Methods

### 2.1. Clinical History and Specimen Collection

A two-year-old WTD (OV1659) was found experiencing a stillbirth on a farm in Central Florida, on 5 June 2022. An emergency c-section was performed by a veterinarian in the afternoon of the same day. OV1659 was healthy prior to the procedure but deteriorated after two 6-month-old fetuses (OV1660 and OV1661) were removed, and the doe was euthanized. A whole blood (WB) specimen from OV1659 was collected by the owner into a 3 mL BD Vacutainer EDTA tube (Becton Dickinson, Franklin Lakes, NJ, USA). The University of Florida (UF) Cervidae Health Research Initiative (CHeRI) team performed necropsies on the two fetuses on the morning of 6 June 2022. During the necropsy, photos of the animals and each specimen were documented. Fresh hepatic tissues (HT), kidney tissues (KT), placenta tissue (PT), and spleen tissues (ST) were collected from both animals. Tissue specimens were stored in 5 mL snap-cap Eppendorf tubes (Thermo Fisher Scientific, Waltham, MA, USA). All specimens were kept on ice immediately after collection and were stored at −80 °C upon arrival at the UF College of Veterinary Medicine laboratory for virology tests at a later time.

### 2.2. Mycobacterium PCR, Toxoplasma gondii PCR, and BTV & EHDV RT-qPCR Detection

Tissue homogenates were prepared by bead-beating as previously described [27]. Total DNA was extracted from the PT homogenates of OV1660 and OV1661 using a DNeasy Mini kit (Qiagen, Valencia, CA, USA) following the manufacturer’s protocol. Total RNA was extracted from the WB of OV1659, HT and ST homogenates of OV1660, and ST homogenates of OV1661 using a QIAamp Viral RNA Mini kit (Qiagen, Valencia, CA, USA) following the manufacturer’s protocol. *Mycobacterium* PCR, *Toxoplasma gondii* PCR, and bluetongue virus (BTV) and epizootic hemorrhagic disease virus (EHDV) RT-qPCR were conducted for differential diagnosis. Placenta tissue homogenates from OV1660 and OV1661 were tested for *Mycobacterium* species using PCR following a previously described protocol [28]. The primers utilized were KY18 (5′-CAC ATG CAA GTC GAA CGG AAA GG-3′) and KY75 (5′-GCC CGT ATC GCC CGC ACG CTC ACA-3′). Additionally, PT homogenates from OV1660 and OV1661 were tested for *Toxoplasma gondii* using nested PCR [29]. The nested PCR was performed using two pairs of primers, external primer set (5′-CGA AAT GGG AAG TTT TGT GAA-3′ and 5′-TTG CGC GAG CCA AGA CAT C-3′) and internal primer set (5′-TGA ATC CCA AGC AAA ACA-3′ and 5′-GCG CGA GCC AAG ACA TCC AT-3′). Furthermore, ST homogenates from OV1660 and OV1661 underwent RT-qPCR targeting BTV and EHDV, as previously described [27].

### 2.3. Cell Culture

Virus isolation from OV1660 and OV1661 hepatic, kidney, and spleen tissue homogenates was attempted in Vero E6 cells (*Cercopithecus aethiops* [African green monkey] (ATCC, Manassas, VA, USA, Cat#: ATCC CRL1586) and BT cells (*Bos taurus [Cow] Cat*#: ATCC CRL1390) obtained from the American Type Culture Collection (ATCC). The cells were propagated as monolayers in 25 cm^2^ vented tissue culture flasks (25 cm^2^ flask, Corning Inc., Corning, NY, USA) using Advanced Dulbecco’s Modified Eagle’s Medium (aDMEM, Thermo Fisher Scientific, Waltham, MA, USA) supplemented with 2 mM L-alanyl-L-glutamine (GlutaMAXTM, Invitrogen Corp., Carlsbad, CA, USA), antibiotics (PSN; 50 μg/mL penicillin, 50 μg/mL streptomycin, 100 μg/mL neomycin [Invitrogen Corp., Carlsbad, CA, USA]) and 10% low-antibody, heat-inactivated, gamma-irradiated fetal bovine serum (FBS, manufacturer certified BVDV negative through florescent antibody [Hyclone, GE, Healthcare Life Sciences, Pittsburgh, PA, USA]). Vero E6 and BT cells were incubated at 37 °C in 5% CO_2_ atmospheres within humidified incubators.

### 2.4. Virus Isolation in Cell Culture

After thawing on ice, aliquots of kidney, liver, and spleen tissue were homogenized using sterile tissue grinders (Covidien, Dublin, Ireland) in 1 mL of phosphate-buffered saline (PBS) (1X, Gibco, Thermo Fisher Scientific, Waltham, MA, USA). A total of 50 µL of the tissue homogenates was added to 3 mL of supplemented aDMEM and filtered through a 0.45 µm pore-size syringe-tip filter (Grainger, Lake Forest, IL, USA) to remove contaminating bacteria and fungi. The resulting filtrates were then used to inoculate confluent monolayers of Vero E6 and BT in 25 cm^2^ vented tissue culture flasks (Corning Inc., Dublin, Ireland). Mock-inoculated cells were maintained in parallel with the inoculated flasks. The inoculated cells were monitored for the formation of virus-induced cytopathic effects (CPE) using an inverted microscope with phase-contrast optics, with refeeds of the cells performed every 4 days, approximately, using FBS media (3% FBS, and the same other components). Aliquots of the spent cell culture media of cells displaying CPE were collected and stored at −80 °C for follow-up analyses at a future time.

### 2.5. BVDV-1 RT-PCR

The spent cell culture media of the BT and Vero E6 cells inoculated with kidney, liver, and spleen were chosen for analyses, based on the presence of virus-induced CPE. After thawing on ice, RNA was extracted from the virions in the spent growth media using a QIAamp Viral RNA Mini Kit (Qiagen, Valencia, CA, USA) according to the manufacturer’s protocol.

The BVDV-1 RT-PCR primer set was designed by the UF CHeRI team using the Primer3 software v4.1.0 [30]. The primer set (forward primer: 5′-GCC TTC TGT GAA AGT ACG GG-3′ and reverse primer: 5′-GGC TGC TGT GAA AGT ACC AG-3′) was obtained from Eurofins Scientific (Louisville, KY, USA) with the expected amplicon size of 400 bp. Conventional RT-PCR was conducted in 30 µL reaction mixtures composed of 6 µL of 5× buffer solution, 4.8 µL of RNA template (up to 100 ng per reaction), 1.2 µL of 10 mM dNTP mix, 6 µL of 5× Q solution (Qiagen, Valencia, CA, USA), 1.2 µL of each primer from 20 mM stocks, 8.4 µL of RNase-free water, and 1.2 µL of RT-PCR enzyme mix (Qiagen, Valencia, CA, USA). Thermocycling was performed in a SimpliAmp thermal cycler (Applied Biosystems, San Francisco, CA, USA) as follows: initial denaturation at 50 °C for 30 min and 95 °C for 5 min, 50 cycles of denaturation for 30 s at 95 °C, annealing for 30 s at 51 °C primer, and elongation at 72 °C for 1 min, final elongation at 72 °C for 30 s, then 72 °C for 7 min follow by 4 °C for ∞. Negative PCR controls were included in each PCR run.The RT-PCR products were analyzed using electrophoresis on a 1.5% molecular biology grade agarose gel (Genesee Scientific, Cajon, CA, USA) prepared with 1X TBE buffer (Thermo Fisher Scientific, Waltham, MA, USA) and stained with ethidium bromide (Thermo Fisher Scientific, Waltham, MA, USA). Each lane contained 25 μL of RT-PCR product mixed with 5 μL of 6X orange DNA loading dye (Thermo Fisher Scientific, Waltham, MA, USA). Electrophoresis was performed using an Owl B2 EasyCast Mini Gel Horizontal Electrophoresis System (Thermo Fisher Scientific, Waltham, MA, USA) with FB300 Electrophoresis Power Supply (Thermo Fisher Scientific, Waltham, MA, USA) under 150 V for 40 min. The PCR results were visualized with an ENDURO GDS TOUCH gel documentation system (Labnet International, Inc., Edison, NJ, USA). The samples tested for BVDV-1 RT-PCR are listed in Table 1.

### 2.6. Sanger Sequencing

The BVDV-1 RT-PCR amplicon products of Vero E6 cells inoculated with liver homogenate from animal OV1660 were purified using the QIAquick gel extraction kit (Qiagen, Valencia, CA, USA). The concentrations of purified amplicons were measured using a Qubit 3.0 (Life Technologies, Carlsbad, CA, USA) with HS DNA reagent. Purified amplicons were then submitted to Functional Biosciences (Madison, WI, USA) for Sanger sequencing. The sequences were assembled using CLC Genomics Workbench v2.0 (Qiagen, Valencia, CA, USA) and subjected to BLASTN (https://blast.ncbi.nlm.nih.gov/Blast.cgi, accessed on 1 June 2025) searches against the National Center for Biotechnology Information (NCBI) non-redundant nucleotide database.

## 3. Results

### 3.1. Gross Observations

**Figure 1:**
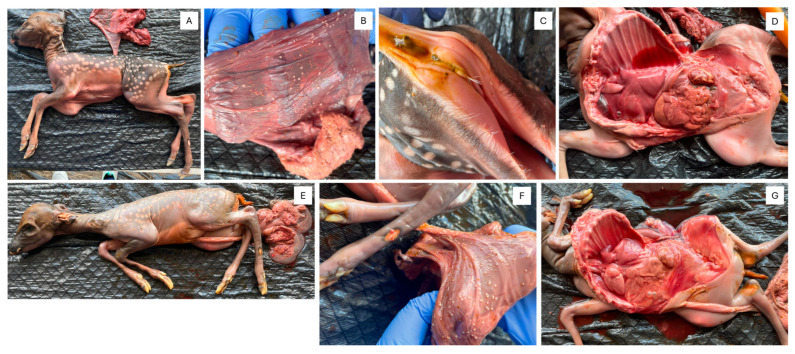
Gross observation of OV1660 (**A**) full body, (**B**) small white nodules indicative of amnion nodosum on the placenta, (**C**) anus with diarrhea, (**D**) full body after removing the rib cage, and OV1661 (**E**) full body, (**F**) small white nodules indicative of amnion nodosum on the placenta, (**G**) full body after removing the rib cage.

Necropsy findings revealed two partially mummified fetuses (OV1660 and OV1661) with underdeveloped eyes, signs of diarrhea, and white, approximately 0.5 cm nodules on their placentas (Figure 1). Fetus OV1660 exhibited hemorrhage within the thoracic cavity. Additionally, its lung tissue sank in water, suggesting the fetus did not breathe post-delivery and likely died in utero prior to stillbirth.

### 3.2. Mycobacterium PCR, Toxoplasma gondii PCR, and BTV & EHDV RT-qPCR Detection

The placenta tissues from both fetuses tested negative for *Mycobacterium* PCR and *Toxoplasma gondii* PCR. The spleen tissues from both fetuses also tested negative for BTV and EHDV by RT-qPCR.

### 3.3. Evidence of Virus Isolation in Cultured Cells

**Figure 2:**
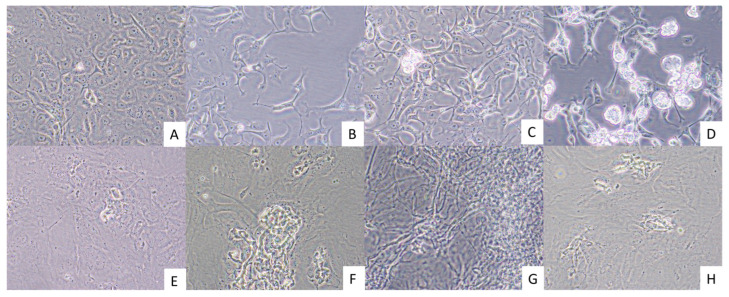
Cytopathic effects in Vero E6 and BT cells inoculated with tissue homogenates from animals OV1660 and OV1661. Vero cells were photographed 18 days post inoculation. (**A**) Mock inoculated Vero E6 cells, (**B**) Vero E6 cells inoculated with OV1660 spleen homogenate, (**C**) Vero E6 cells inoculated with OV1660 liver homogenate, and (**D**) Vero E6 cells inoculated with OV1661 kidney homogenate. BT cells were photographed 27 days post-inoculation. (**E**) Mock inoculated BT cells, (**F**) BT cells inoculated with OV1660 liver homogenate, (**G**) BT cells inoculated with OV1661 liver homogenate, and (**H**) BT cells inoculated with OV1661 spleen homogenate. Images were captured at 400× magnification.

Virus-induced CPE were observed in Vero E6 cells by 18 days post-inoculation (dpi) of the cells with HT, KT, and ST homogenates (Figure 2B–D). In comparison, virus-induced CPE were present much later in the BT cells, 27 dpi, and were not as obvious as those observed within Vero E6 cells (Figure 2F–H). The CPE included darkening of the cell cytoplasm followed by detachment of dead cells from the growing surface of the cell culture flasks.

### 3.4. BVDV-1 RT-PCR

The whole blood sample from doe OV1659, Vero E6 cell cultures inoculated with OV1660 spleen and liver tissues, BT cell cultures inoculated with OV1660 kidney and liver tissues, and BT cells inoculated with OV1661 kidney, liver, and spleen tissues were positive for BVDV-1 by RT-PCR. The liver and spleen tissues from OV1660, BT cells inoculated with OV1660 spleen tissue homogenate, and the spleen tissue from OV1661 tested negative for BVDV-1 RT-PCR. The results of RT-PCR tests are given in Table 1. Electrophoresis gel images of BVDV1 RT-PCR results are presented in Figure S1.

### 3.5. Sanger Sequencing

Following quality trimming and assembly, the amplicon products of cell culture Vero E6 inoculated with liver homogenates from animals OV1660 yielded 400 bp. BLASTN analysis showed 96.75% identity (100% coverage) to BVDV-1 strain Chilgok (GenBank accession number: ON676187) in the NCBI GenBank database. The BVDV-1 amplicon sequence from OV1660 is available under the NCBI GenBank accession number PV548932.

## 4. Discussion

To our knowledge, this study documents the first confirmed case of naturally occurring BVDV-1 infection associated with fetal mummification in a farmed WTD. The molecular detection of BVDV-1 RNA in both maternal and fetal specimens, in conjunction with virus isolation from multiple fetal tissues, provides compelling evidence of vertical transmission, a known outcome of BVDV infection during early gestation in cattle and previously demonstrated under experimental conditions in WTD. The gross pathology findings, including fetal death, mummification and underdeveloped eyes, are consistent with prior descriptions of BVDV-induced reproductive failure in WTD [17,19,20].

The RT-PCR assay developed in this study provided a sensitive and specific method for detecting BVDV-1 RNA from whole blood samples and tissue culture supernatants. This finding highlights the value of combining molecular diagnostics with virus culture techniques to improve detection sensitivity. Differential diagnostic testing ruled out other viral and protozoal causes of fetal mummification in this case, strengthening the association of BVDV-1 as the primary etiological agent. The BVDV-1 amplicon sequence from OV1660 showed high similarity (96.75%) to the Chilgok strain from cattle in South Korea, a finding that may warrant further investigation into strain origin, movement, and evolutionary dynamics in farmed cervid populations in North America. Future studies should include serology tests to gain insights on the immunological tolerance status among the deer to BVDV, and complete virus genome sequence analyses to better understand the prevalence of specific strains and the disease dynamics within a herd.

In conclusion, this report expands the clinical and molecular understanding of BVDV-1 in non-bovine hosts and provides practical diagnostic tools that may enhance future diagnostic and surveillance efforts. Our findings underscore the importance of using biosecurity measures and including BVDV in differential diagnosis of abortions to reduce the risk of BVDV transmission and potential outbreaks, particularly during early gestation.

## Figures and Tables

**Table 1:** Summary of the results of PCR-based tests and Sanger sequencing.

Sample ID	*Mycobacterium* PCR	*Toxoplasma gondii* PCR	BTV and EHDV RT-qPCR	BVDV1 RT-PCR	Sanger Sequencing
OV1659-WB	-	-	Negative	Positive	-
OV1660-ST	-	-	Negative	Negative	-
OV1660-HT	-	-	-	Negative	-
OV1660-PT	Negative	Negative	-	-	-
OV1660-ST_Vero E6	-	-	-	Positive	-
OV1660-HT_Vero E6	-	-	-	Positive	BVDV-1
OV1660-ST_BT	-	-	-	Negative	-
OV1660-HT_BT	-	-	-	Positive	-
OV1660-KT_BT	-	-	-	Positive	-
OV1661-ST	-	-	Negative	Negative	-
OV1661-PT	Negative	Negative	-	-	-
OV1661-ST_BT	-	-	-	Positive	-
OV1661-HT_BT	-	-	-	Positive	-
OV1661-KT_BT	-	-	-	Positive	-

WB: whole blood; ST: spleen tissue; HT: liver tissue; PT: placenta tissue; KT: kidney tissue; BT: bovine turbinate cell culture; Vero E6: African green monkey kidney epithelial cell culture; BTV: bluetongue virus; EHDV: epizootic hemorrhagic disease virus; BVDV: bovine viral diarrhea virus; -: not tested.

## Data Availability

The amplicon sequence in this study has been deposited in the NCBI GenBank database and is available under the NCBI GenBank accession number PV548932.

## Supplementary Materials

The following supporting information can be downloaded at: https://www.mdpi.com/article/10.3390/v17081104/s1. Figure S1. Electrophoresis gel images of BVDV1 RT-PCR results. A: OV1659-WB; B: OV1660-ST; C: OV1660-HT; D: OV1660-ST_Vero E6; E: OV1660-HT_Vero E6; F: OV1660-ST_BT; G: OV1660-HT_BT; H: OV1660-KT_BT; I: OV1661-ST; J: OV1661-ST_BT; K: OV1661-HT_BT; L: OV1661-KT_BT; NC: PCR negative control. WB: whole blood; ST: spleen tissue; HT: liver tissue; KT: kidney tissue; BT: bovine turbinate cell culture; Vero E6: African green monkey kidney epithelial cell culture.

## Abbreviations

The following abbreviations are used in this manuscript:

ATCC	American Type Culture Collection
BTV	Bluetongue virus
BVDV	Bovine viral diarrhea virus
dpi	Day post-inoculation
CHeRI	Cervidae Health Research Initiative
CP	Cytopathic
CPE	Cytopathic effects
EHDV	Epizootic hemorrhagic disease virus
FBS	Fetal bovine serum
HT	Hepatic tissues
KT	Kidney tissues
NCBI	National Center for Biotechnology Information
NCP	Non-cytopathic
PBS	Phosphate-buffered saline
PI	Persistently infected
PT	Placenta tissue
RT-PCR	Reverse transcription PCR
ST	Spleen tissues
UF	University of Florida
WB	Whole blood
WTD	White-tailed deer
