# Supplementary figures and images for: Bovine Viral Diarrhea Virus-1 (Pestivirus bovis) Associated with Stillborn and Mummified Fetuses in Farmed White-Tailed Deer (Odocoileus virginianus) in Florida
Source: Viruses. 2025 Aug 12;17(8):1104. doi: 10.3390/v17081104 (PMC12390654; doi:10.3390/v17081104)

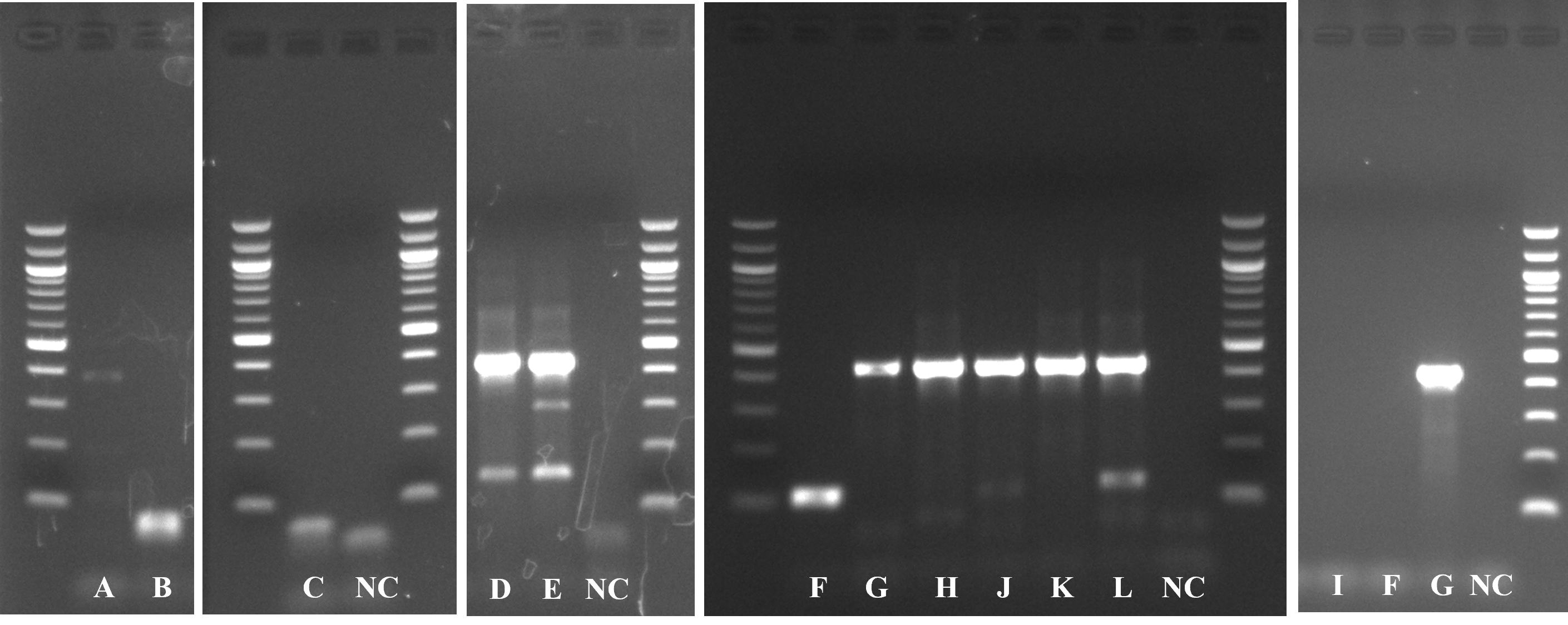

Supplement: Supplementary file 1 [file viruses-17-01104-s001.zip › viruses-3714420-supplementary.tif]
